# Champuru 2: Improved Scoring of Alignments and a User‐Friendly Graphical Interface

**DOI:** 10.1111/1755-0998.70110

**Published:** 2026-04-26

**Authors:** Yann Spöri, Jean‐François Flot

**Affiliations:** ^1^ Evolutionary Biology & Ecology Université libre de Bruxelles (ULB) Brussels Belgium; ^2^ Interuniversity Institute of Bioinformatics in Brussels – (IB)² Brussels Belgium; ^3^ Brussels Laboratory of the Universe – BLU Brussels Belgium

**Keywords:** bioinformatics, chromatogram analysis, double peaks, Haxe, heterozygosity, mixed traces, Sanger sequencing, transpiling

## Abstract

Champuru is a web‐based software tool that helps determine the two sequences present in mixed Sanger chromatograms obtained by simultaneously sequencing two DNA templates of unequal lengths. A previous version (Champuru 1.0) was published as a simple Perl CGI (Common Gateway Interface) application, but the server hosting it was decommissioned, which prompted us to update Champuru and develop it further. The new Champuru 2, implemented in Haxe and hosted at GitHub Pages, offers an improved graphical user interface as well as more sophisticated algorithms to compute alignment scores, making it more efficient at detecting the most likely alignment positions between forward and reverse traces. It also compares the distribution of alignment scores to the theoretical expectation for the comparison of two random sequences and uses this comparison to calculate *p*‐values for the offset pairs it detects. Moreover, Champuru 2 now makes it possible to analyse other offset pairs than the one detected as most likely by the selected algorithm. Champuru 2 is freely accessible at https://eeg‐ebe.github.io/Champuru/, including both a graphical user interface (running a JavaScript version transpiled from the Haxe source code) and a compiled command‐line version (obtained by transpiling the Haxe source code into C++).

## 1 Introduction

Although Sanger sequencing is becoming progressively superseded by the rise of next‐generation sequencing methods, it is still useful when it comes to small‐scale projects such as testing for the presence of cryptic species (Sporre et al. 2024) or checking the result of a genome‐editing experiment (Rausch et al. 2020). However, one limitation of Sanger sequencing (as for all first‐generation sequencing methods) is that, when applied to mixtures of DNA templates (e.g., when PCR‐amplifying nuclear markers of diploid organisms), it results in double peaks, the phasing of which is often difficult (Harrigan et al. 2008; Browning and Browning 2011; Flot et al. 2006).

To help alleviate this issue, the program Champuru was published in 2007 to deconvolute mixed traces obtained by sequencing a mixture of two DNA sequences of uneven lengths (Flot 2007). Champuru takes advantage of the fact that the double peaks in the chromatograms of length‐variant heterozygous (LVH) individuals are different in the forward and reverse directions (Flot et al. 2006). As a result, the information contained in the forward and reverse chromatograms can be combined and used to reconstruct the sequences of both haplotypes, in a process akin to solving a system of two equations with two unknowns (Flot et al. 2006).

In contrast to other programs that either attempt to guess the two haplotypes from a single chromatogram but often do not resolve them completely, such as ShiftDetector (Seroussi et al. 2002), In*d*elligent (Dmitriev and Rakitov 2008) and CHILD (Zhidkov et al. 2011); need prior knowledge of the possible variants, such as TraceHaplotyper (Seroussi and Seroussi 2007) and BCV (Fantin et al. 2013); require a reference sequence, such as Mixed Sequence Reader (Chang et al. 2012) and CRISP‐ID (Dehairs et al. 2016); or leverage population‐level information to perform statistical phasing, such as SeqPHASE (Flot 2010; Spöri and Flot 2024), Champuru uses information from both the forward and reverse chromatograms of a mixed amplicon to reconstruct its two constituent sequences with certainty, without requiring any additional information (Flot et al. 2006; Flot 2007). It does so by establishing a one‐to‐one relationship between the two input strings representing the forward and reverse chromatograms on the one hand and the two output strings corresponding to the two deconvoluted haplotypes on the other hand. The process starts by comparing the forward and reverse input strings in order to find their two matching alignment positions: unlike length‐variant homozygous individuals (the forward and reverse chromatograms of which can only align in a single way), the chromatograms of length‐variant heterozygous individuals can be aligned in two possible ways, the consensus of each of which corresponds to one of their two haplotypes (Flot et al. 2006). Champuru then calculates these two consensuses, and solves any remaining ambiguity by assuming that there are only two haplotypes in the mixture and that therefore any peak in the initial chromatograms that is not accounted for by one haplotype necessarily comes from the other one (Flot 2007). Finally, as input strings often contain basecalling errors, Champuru checks that the result of sequencing a mixture of the two haplotypes it has reconstructed does explain all the ambiguities in the input strings, emitting a warning every time it detects a potential problem. In such cases, users are then invited to check their chromatograms at the listed positions and resubmit corrected input sequences, until no problem is detected (Flot 2007).

This iterative, user‐interactive approach sets Champuru apart from all other programs mentioned above, as does the fact that Champuru uses information obtained from sequencing PCR products in both the forward and reverse directions. As it occupies a specific niche among programs aimed at disentangling mixed traces, Champuru has been used in many studies, with a total of 138 citations in Google Scholar as of 15 August 2025. However, Champuru 1.0 was written in Perl with CGI (Common Gateway Interface; Shishir 1996): such design is not optimal, as it requires a dedicated server to which users' input sequences are uploaded and from where the result of the analysis is downloaded, which entails maintenance costs and security issues. For this reason, we reimplemented Champuru into an updated version, Champuru 2, with improved scoring alignments, additional statistical tests and a user‐friendly graphical interface. Champuru 2 was written in Haxe (Spöri and Flot 2024) and is accessible online at https://eeg‐ebe.github.io/Champuru.

## 2 Material and Methods

### 2.1 Input

As with Champuru 1.0, Champuru 2 cannot process directly chromatogram files but takes as input two strings describing the base calls of the forward and reverse chromatograms, respectively. These strings can be generated using a variety of trace‐processing software (Tippmann 2004), such as the open‐source programs Phred (Ewing et al. 1998; Ewing and Green 1998), BioEdit (Hall 1999), SeqTrace (Stucky 2012), the Staden package (Staden 1996) and TraceTuner (Struck et al. 2012); the freeware tools FinchTV (Geospiza), Chromas (Technelysium), 4Peaks (Nucleobytes) and MEGA (Tamura et al. 2021); or the commercial programs ChromasPro (Technelysium), CodonCode Aligner (CodonCode Corporation; Richterich 2004) and Sequencher (GeneCodes; Nishimura 2000). Each character of the input strings corresponds to a single or double peak in the corresponding forward or reverse chromatograms using the one‐letter codes of the 1984 recommendations of the Nomenclature Committee of the International Union of Biochemistry (Cornish‐Bowden 1985). Because some of the programs listed above can only process traces one by one, without automatically reverse‐complementing reverse chromatograms, a button located right under the “reverse sequence” field allows users to reverse‐complement this sequence if it was entered in the 5’→3′ orientation.

### 2.2 Step 1—Alignment Score Calculation

Champuru first calculates alignment scores for all possible alignments of the two input strings in order to find the best offset positions (the offset being defined as the position of the first base of the forward sequence by reference to the first base of the reverse sequence; Flot 2007). Champuru 2 now allows users to choose among three possible scoring schemes:

1. *overlap‐corrected score*, which is the same alignment scoring algorithm already described in the original Champuru paper (namely, computing the number of compatible positions in the alignment minus one fourth of the length of the overlap between the two sequences, to correct for the fact that two random nucleotides have a 0.25 probability of matching just by chance);
2. *overlap and ambiguity‐corrected score*, a modification of the scoring scheme that accounts for the fact that ambiguous characters (e.g., W) can match multiple characters (namely A, T, K, W, R, M, Y, B, D, H, V and N), therefore attributing a score of 0.5 (instead of 1) to any such match;
3. *longest uninterrupted overlap score*, a novel scoring scheme that returns the length of the longest stretch of consecutive matching nucleotides as a score.

**FIGURE 1:**
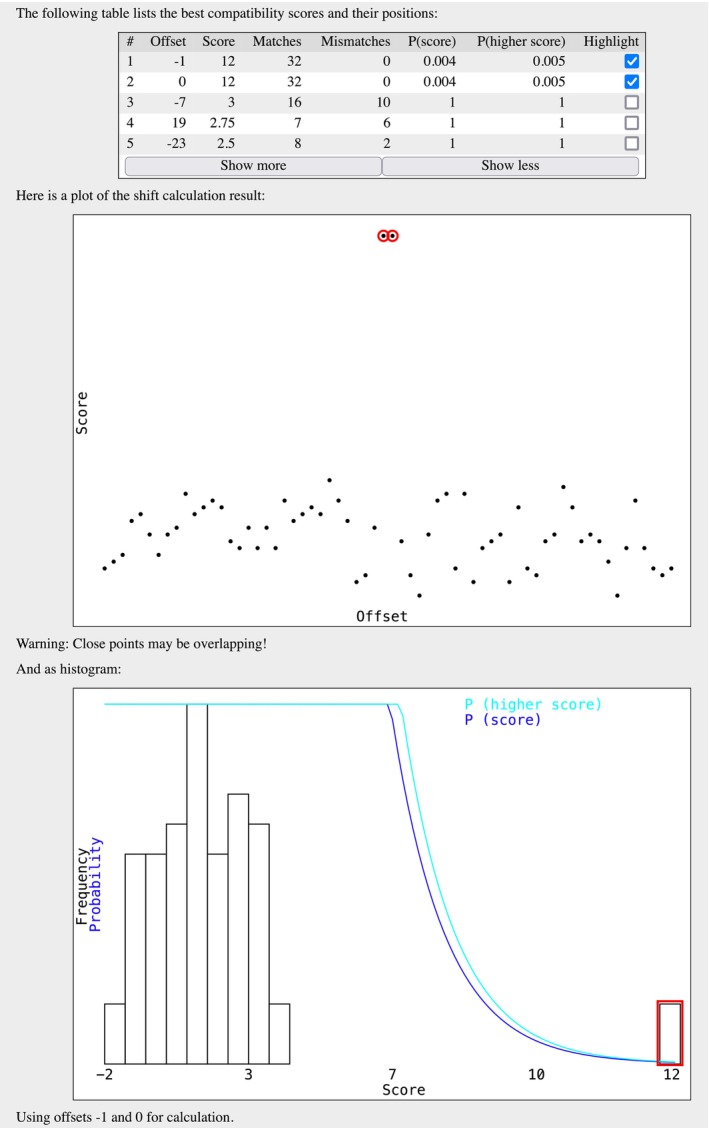
Graphical output of Champuru 2 for the sequence pair CTRAATTCAAATCACACTCGCGAAAWYMWKRAA (forward) and CYWRAWTYMAAWYMMMMYYSSSRAAATCATGAA (reverse) using the default scoring scheme. First, the best alignment scores are displayed in a table as well as in a scatter plot, allowing users to judge how many alignment positions to consider for downstream analyses. These alignment scores are also displayed in a scatter plot, followed by a histogram comparing the observed score distribution with the null hypothesis of a Gumbel distribution, allowing users to detect statistically significant outliers.

Champuru 2 sorts the alignments according to their alignment score and—in case of equal alignment scores—according to the number of observable mismatches. Then it displays the five best alignments in a table and produces a scatter plot of the alignment score against the offset between the two sequences (Figure 1). Champuru 2 also produces a histogram showing the distribution of alignment scores, with two curves superimposed on it: one showing the theoretical probability density of alignment scores under the null hypothesis of random sequences, and another curve showing the corresponding tail function (or complementary cumulative distribution function; i.e., the theoretical probability of observing an alignment with a score higher than a given value). To calculate these curves, it takes advantage of the classical result that the probability distribution of ungapped alignment scores of two random sequences is an extreme value type I distribution, also known as a Gumbel distribution (Gumbel 1935; Karlin and Altschul 1990; Ortet and Bastien 2010). To estimate this null distribution, Champuru 2 shuffles the characters in the input sequences 2000 times, each time picking up one offset randomly and calculating the corresponding alignment score. The mean of the observed alignment scores as well as the observed standard deviation are then fitted to a Gumbel distribution. This allows users to visualise whether there are outliers in the alignment scores distributions, i.e., offsets for which the alignment score is much higher than expected following the null distribution. By clicking on a dot in the scatter plot or on a box in the histogram, users can access further information about the data. Also by hovering the mouse over a line in the table (and/or by ticking a checkbox on the right side of the table), the corresponding points in the alignment vs. offset score plot and in the histogram become highlighted, allowing users to better grasp the correspondence between the three.

**FIGURE 2:**
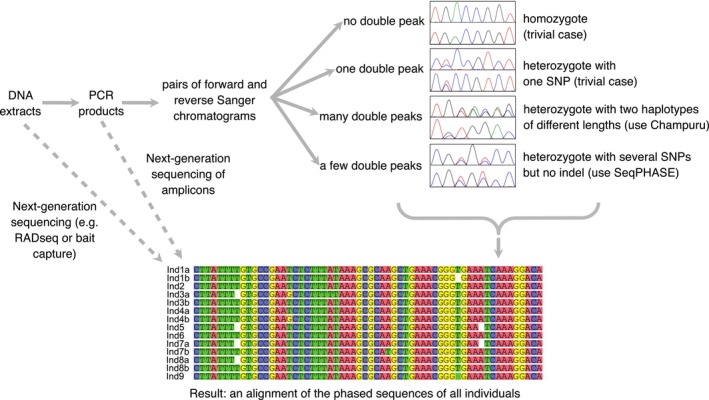
General flow chart showing the process for generating an alignment of phased sequences.

**FIGURE 3:**
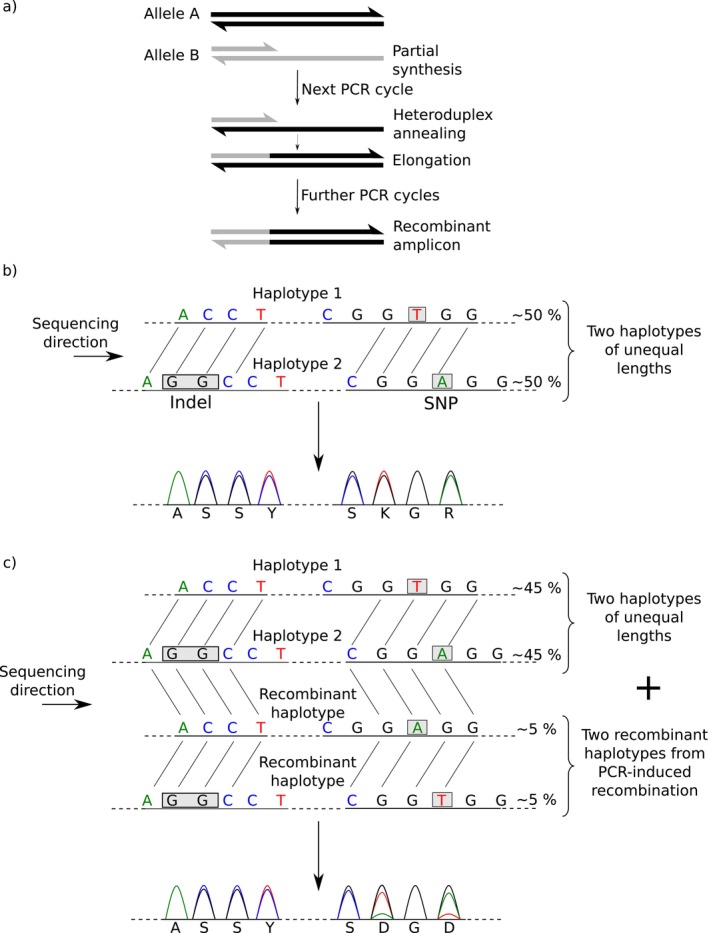
Schematic representation of how PCR‐induced recombination between two haplotypes of different lengths can result in additional, artefactual haplotypes that show up as triple peaks in Sanger chromatograms (modified from Flot et al. 2018).

Four possible situations can happen:

1. no clear outlier is identified in the distribution of observed scores when compared to the null distribution. This means that the forward and reverse sequences do not align with each other better than a pair of random sequences of similar base composition, suggesting that there is a problem with the input data (for instance, the forward and reverse input sequences do not match);
2. a single outlier is found, indicating that there is only one way to align the forward and reverse sequences with one another. This suggests that the individual sequenced was homozygous at this marker (in which case double peaks observed in the forward and/or reverse chromatogram sequences are mere basecalling errors), or that it was heterozygous but with two haplotypes of equal lengths (in which case the observed double peaks are expected to be identical between the forward and reverse sequences). For phasing heterozygous individuals with several double peaks but no heterozygous indel, users may use the Bayesian phasing approach implemented in SeqPHASE (Flot 2010; Spöri and Flot 2024) (Figure 2);
3. two clear outliers in the score distribution are identified, corresponding each to one possible alignment position. This is the expected outcome for length‐variant heterozygotes, which allows reconstructing their two haplotypes with near‐certainty by combining the information contained in the forward and reverse chromatograms (Flot et al. 2006);
4. more than two outliers are detected. This means that there are more than two possible ways to align the provided forward and reverse input sequences, indicating that the corresponding amplicon contained more than two haplotypes of different lengths. This can point to copy‐number variation or whole‐genome duplication in the target genome (Flot et al. 2008) or may result from experimental artefacts such as contamination or aspecific amplification during PCR; in addition, PCR‐mediated recombination (Judo et al. 1998) can sometimes produce additional, artefactual haplotypes that result in similar patterns (Figure 3). In such situations, the information contained in the forward and reverse string is usually not sufficient to reconstruct the various haplotypes with certainty. Still, comparison of partially reconstructed haplotypes (obtained for various offset pairs) with haplotypes found in other individuals in the same population often makes it possible to guess the most likely haplotypes present in such mixtures. To facilitate such guesswork, Champuru 2 now allows users to navigate among different offset pairs: they can enter their desired offsets using the button *Use different offsets* or alternatively may rerun the analysing after ticking the checkbox *Analyze further offset pairs*, which produces a table listing all detected outlier offset pairs at the very bottom of the page.

At the end of Step 1, users can download a table containing the alignment scores and their associated probabilities. For the following steps, Champuru 2 uses by default the two offsets corresponding to the alignments with the highest scores.

### 2.3 Step 2—Consensus Sequence Calculation

As a second step, Champuru calculates consensus sequences for the two input sequences in the two offset positions determined in Step 1. In contrast to Champuru 1.0, which only considered positions for which information from both input strings was available, Champuru 2 also displays (but in lowercase) the positions in the consensuses that are only supported by one of the two input sequences.

### 2.4 Step 3—Sequence Reconstruction

In the simplest case when the two haplotypes differ by a single heterozygous indel and a few single‐nucleotide polymorphisms (SNPs), consensus sequence calculation is enough to recover the two haplotypes without ambiguities left. However, in more complex cases some ambiguities in the consensus sequences remain unresolved, and Step 3 solves them by considering that any peak detected in the forward or reverse basecalled chromatogram strings has to come from one haplotype or the other. As an example, to find out whether a Y in a consensus sequence is a C or a T, Champuru traces this Y back to matching Ys in the forward and reverse basecalled chromatograms strings and checks whether their Cs or Ts are already explained by the other haplotype (corresponding to the other offset position). For instance, if a Y at a position in one input sequence corresponds to a C in one haplotype, then the other haplotype has to have a T at the corresponding site (Flot et al. 2006).

### 2.5 Step 4—Checking Sequences

Finally, the sequences reconstructed in Step 3 are checked for consistency with the initial input strings (by verifying whether the simultaneous sequencing of a mixture of the two reconstructed haplotypes would yield the pattern of double peaks observed in the forward and reverse chromatograms) as well as with the result of the older Champuru v1.0 perl script.

### 2.6 Step 5—Analysing Further Offset Pairs

In a last, facultative step (only executed when the checkbox *Analyze further offset pairs* is selected) Champuru 2 runs the sequence reconstruction algorithm for a number of best‐scoring offset pairs and displays the number of mismatches for each of them. Users can then click on any specific offset pair to obtain the corresponding results. As this calculation takes extra time, this option is not selected by default.

## 3 Discussion

**FIGURE 4:**
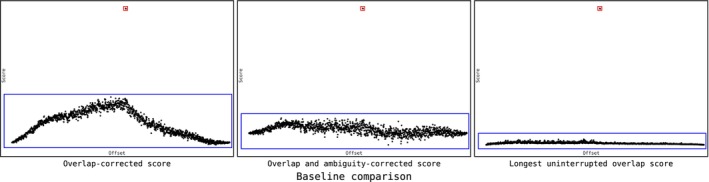
Comparison of the dot plots produced by the three available scoring algorithms on the “real‐life example” provided on the Champuru 2 website.

Figure 4 compares the results of three proposed alignment scoring schemes on a typical set of chromatogram (the ‘real‐life example’ provided on the Champuru 2 website). It reveals that the originally published scoring scheme, with its simplistic approach, presents a ‘mountain‐like’ baseline that peaks at an offset of zero (at the center of the plots), which could lead it to select the wrong offset in case the signal over noise ratio is low. By contrast, the two novel schemes proposed for the first time here have a much flatter baseline, thereby avoiding such bias.

In order to test whether the new scoring schemes are indeed more effective at identifying the correct offsets, we used simulations to compare the performance of the three alignment scoring schemes implemented in Champuru 2. To this aim, we simulated 1000 random sequences with lengths ranging from 25 to 200 bp and with base composition biases ranging from 30% to 50% GC. Each of these 1000 replicate sequences was duplicated to turn it into a pair of haplotypes, to which indels were introduced: in the first haplotype, one base occurring at 1/4 of the length of the sequence was deleted; whereas in the second haplotype, two bases occurring at 3/4 of the length of the sequence were deleted. The resulting two haplotypes had a 1 bp difference in length. We then simulated the result of sequencing a mixture of these two haplotypes starting either from the left (forward sequence) or from the right (reverse sequence). Random mutations were finally introduced in the obtained forward and reverse sequences. In order to mimic basecalling errors, for each position in the simulated chromatogram we started by drawing a random number between 0 and 1: if this random number was higher than the error rate, the position was considered as basecalled properly, whereas if this random number was lower than the error rate, we considered that the position was affected by basecalling uncertainty and replaced the corresponding character by a random draw of one of 16 equally probable possibilities (A, C, T, G, K, M, R, S, W, Y, B, D, H, V, N, or no change). Finally, an extra padding of 0, 100, 200 or 300 random nucleotides was added at the 3′ end of the reverse chromatogram sequence, in order to simulate increasing offset values (as observed in real chromatograms with noisy 3′ ends).

**FIGURE 5:**
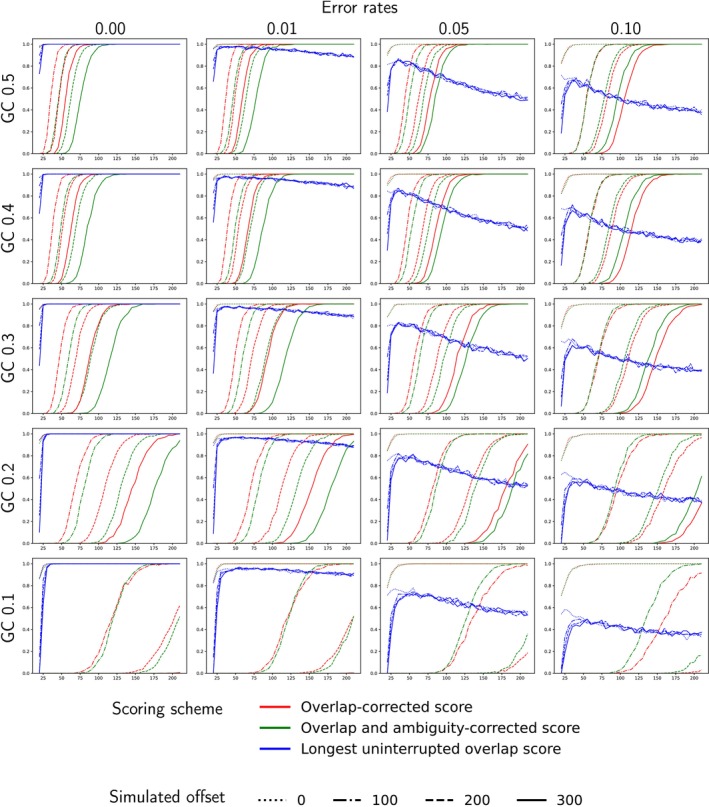
Assessment of the performance of the three proposed scoring schemes using simulations with various lengths of sequences (horizontal axis), offset lengths between the forward and reverse chromatograms (line styles), error rates of the chromatograms (horizontal arrangement of panels), and GC contents of the simulated sequences (vertical arrangement of panels). The vertical axis of each plot indicates the proportion of simulations where the algorithm detected the correct offset.

Results of these simulations (Figure 5) show that the novel ‘Longest uninterrupted overlap’ scoring scheme performs best when sequencing error rates are low, whereas the two other scoring schemes require long sequences to yield good results (especially when error rates and/or simulated offsets are high, or when there is a strong unbalance in nucleotide composition). The ‘Overlap correction’ scoring scheme performs better than the ‘Overlap and ambiguity correction’ scoring scheme when error rates are small (5% and less), whereas ‘Overlap and ambiguity correction’ performs better than ‘Overlap correction’ for high error rates.

Mixed Sanger traces are usually several hundred bases long, under which the ‘Longest uninterrupted overlap’ scoring scheme would perform poorly. Besides, although Sanger sequencing is often described as having a very high accuracy (up to 99.999%; Shendure and Ji 2008), the error rate is many orders of magnitude higher for mixed traces resulting from the simultaneous sequencing of two templates of different lengths (Flot 2007). We therefore recommend the use of the ‘Overlap and ambiguity correction’ scoring scheme by default.

## 4 Conclusion

Compared to the previously published version (Flot 2007), this improved version offers the following enhancements:

1. several scoring schemes for finding the best alignment positions;
2. a series of graphical outputs highlighting the best alignment positions and their respective scores (only in the graphical version of the program);
3. a calculation of the statistical significance of the best scores obtained compared to the null hypothesis of a Gumbel distribution;
4. the possibility of choosing alternative pairs of offsets (instead of solely the best‐scoring offset pair).

Our reimplementation of Champuru using the Haxe language does not only ensure that this useful piece of code is again available to everyone, but also that it is available both as a user‐friendly graphical version and as a command‐line version. Being hosted on GitHub Pages (https://eeg‐ebe.github.io/Champuru), the code runs directly in the web browser of the user on their own computer, eliminating the hurdle of maintaining an online, secure server and also ensuring that users' sequence data are not transmitted over the internet. Compiled C++ versions for linux and MacOS are provided for download (https://eeg‐ebe.github.io/Champuru/download.html) but since the original Haxe code is available at https://github.com/eeg‐ebe/Champuru, interested users may also transpile it into a variety of other languages such as Neko, Python etc. if they wish.

As several improvements to the code were introduced in this new version, for reverse compatibility Champuru 2 starts by calculating the output of the previous version (1.0) of Champuru. In case a mismatch is detected with the result of the new version, a warning message is displayed inviting the user to contact the authors.

## Conflicts of Interest

The authors declare no conflicts of interest.

## Data Availability

Data sharing not applicable to this article as no datasets were generated or analysed during the current study.
